# What are the short-term annual cost savings associated with kidney transplantation?

**DOI:** 10.1186/s12962-022-00355-2

**Published:** 2022-05-03

**Authors:** Prosper Koto, Karthik Tennankore, Amanda Vinson, Kristina Krmpotic, Matthew J. Weiss, Chris Theriault, Stephen Beed

**Affiliations:** 1 Research Methods Unit, Nova Scotia Health, 5790 University Avenue, Halifax, NS B3H 1V7 Canada; 2 Department of Medicine (Division of Nephrology), Dalhousie University, Halifax, NS Canada; 3 Department of Critical Care, Dalhousie University, Halifax, NS Canada; 4 Centre Mère-Enfant Soleil du CHU de Québec, Transplant Québec, Québec, QC Canada; 5 Research Methods Unit, Nova Scotia Health, Halifax, NS Canada; 6 Department of Critical Care, Department of Anesthesia, Pain Management and Perioperative Medicine, Dalhousie University, Halifax, NS Canada

**Keywords:** Kidney transplantation, High-cost, Cost savings, Cost-group, Finite mixture model

## Abstract

**Background:**

Kidney transplantation (KT) is often reported in the literature as associated with cost savings. However, existing studies differ in their choice of comparator, follow-up period, and the study perspective. Also, there may be unobservable heterogeneity in health care costs in the patient population which may divide the population into groups with differences in cost distributions. This study estimates the cost savings associated with KT from a payer perspective and identifies and characterizes both high and low patient cost groups.

**Method:**

The current study was a population-based retrospective before-and-after study. The timespan involved at most three years before and after KT. The sample included end-stage kidney disease patients in Nova Scotia, a province in Canada, who had a single KT between January 1, 2011, and December 31, 2018. Each patient served as their control. The primary outcome measure was total annual health care costs. We estimated cost savings using unadjusted and adjusted models, stratifying the analyses by donor type. We quantified the uncertainty around the estimates using non-parametric and parametric bootstrapping. We also used finite mixture models to identify data-driven cost groups based on patients’ pre-transplantation annual inpatient costs.

**Results:**

The mean annual cost savings per patient associated with KT was $19,589 (95% CI: $14,013, $23,397). KT was associated with a 24–29% decrease in mean annual health care costs per patient compared with the annual costs before KT. We identified and characterized patients in three cost groups made of 2.9% in low-cost (LC), 51.8% in medium-cost (MC) and 45.3% in high-cost (HC). Cost group membership did not change after KT. Comparing costs in each group before and after KT, we found that KT was associated with 17% mean annual cost reductions for the LC group, 24% for the MC group and 26% for the HC group. The HC group included patients more likely to have a higher comorbidity burden (Charlson comorbidity index ≥ 3).

**Conclusions:**

KT was associated with reductions in annual health care costs in the short term, even after accounting for costs incurred during KT.

**Supplementary Information:**

The online version contains supplementary material available at 10.1186/s12962-022-00355-2.

## Introduction

Patients with end-stage kidney disease (ESKD) who qualify and receive kidney transplantation (KT) often enjoy a better quality and length of life than those who do not. However, the effect of KT on health care costs depends on the context. High initial health care costs follow the early period after KT because of the costs associated with organ procurement, surgery, the pre-and post-operative hospital stay, induction agents, and higher doses of maintenance immunosuppression in the early post-transplantation period. However, although subsequent costs after this early period could offset the cost increase during KT [1], the magnitude remains unclear. In addition, previous studies reporting cost savings associated with KT differ in the choice of comparator, the length of the follow-up period, and the adopted study perspective, impacting their generalizability [1, 2].

There may also be unobservable heterogeneity in a given patient population, which may divide the patient population into groups with different cost distributions. These distributions may group patients into high (or low) intensity health resource users among patients with ESKD who received single-kidney transplantation. However, no previous study has identified and characterized patients in cost groups without imposing pre-determined groups to the best of our knowledge. Also, although reporting cost savings, some previous studies utilized long time horizons (≥ 10 years), complicating the attribution of cost savings to KT alone [2–4]. Other studies also examined factors associated with high health care costs without characterizing what constitutes a high cost [1].

Nova Scotia (NS), a province in Canada, changed its organ and tissue donation law on January 18th, 2021. The deemed consent legislation, the Human Organ and Tissue Donation Act (HOTDA), otherwise referred to as the presumed consent model, received Royal Assent on April 12th, 2019, and became effective on January 18th, 2021. The Act includes moving from an opt-in to an opt-out consent model, accompanied by health system transformations. Part of understanding the anticipated effectiveness of the legislation was to examine the potential cost savings associated with KT within the NS health system. Of particular interest would be the impact in the short-term, defined in this study as at most three years before and after KT. The present study aims to quantify the mean annual cost savings associated with KT and identify and characterize patients in data-driven cost groups from a Canadian payer perspective. Understanding the cost savings in NS will allow stakeholders in NS and elsewhere to set reasonable targets for judging the economic impact of deemed consent.

## Methods

### Study design and scope

We utilized a retrospective before-and-after design [2] of NS patients identified through the Multi-Organ Transplant Program (MOTP) database with ESKD who had a single deceased or living-donor KT between January 1, 2011, and December 31, 2018, and health resource use data from January 1, 2010, to December 31, 2019. The overlap in years ensured at least one year before and after KT health resource use data for each patient. Also, we limited the sample to at most three years before and after transplantation. The exclusion criteria consist of non-residency of NS, individuals not covered by the provincial health insurance for medical purposes, recent immigrants who have lived less than a year in NS, self-paid, military families with Canadian Forces health coverage, and individuals who received two KTs within the period given they may not be comparable to those who received solitary transplantation [1]. We used the relevant International Classification of Diseases 10th revision (ICD-10) and the Canadian Classification of Health Interventions (CCI) codes to identify patient and cost elements (Additional file 1: Tables S1 and S2).

We linked NS patients in the MOTP database to health resource use data at the Health Data Nova Scotia (HDNS). The MOTP, located at Queen Elizabeth II (QEII) Health Sciences Centre in Halifax, NS, is the only transplant facility in Atlantic Canada, serving a combined population of 2,446,405 [5] as of the end of 2020, and works collaboratively with other provincial organ and tissue donation programs located in the provinces of New Brunswick, Prince Edward Island, and Newfoundland, to provide kidney, liver, and heart transplantation services and previously, pancreas transplantation. The MOTP has a robust database with information on NS patients with ESKD who are referred for and subsequently undergo KT. The HDNS data repository, located in the Faculty of Medicine's Department of Community Health and Epidemiology at Dalhousie University, NS, provides access to linkable provincial administrative health data sets, including physician billings and hospital discharge abstracts.

### Comparator

The allocation of donated kidneys depends on existing criteria around priority used by the MOTP. These factors include high priority medically sick patients and those requiring multiple organs, recipient sensitization status, dialysis wait time, immunologic compatibility (compatible blood type, human leukocyte antigen match, and presence or absence of donor-specific antibodies), comorbidities, and age [6]. Because there is an increased risk of death or removal from the waitlist among sicker patients, patients who receive a KT may not represent all patients on the waitlist. In addition, their resource utilization (before, during and after KT) may differ from those never transplanted due to greater marginality or comorbidity burden rendering them more likely to be on temporary hold, withdrawn, or die on the waitlist [1, 2]. As a result, we adopted a before-and-after design framework, where each transplantation recipient served as their own control. The difference in the before-and-after costs was the estimated cost savings attributable to KT.

### Patient clinical and socio-demographic characteristics

Patient characteristics included age at transplantation, sex at birth, blood type, and Charlson comorbidity index (CCI) one year before KT, calculated using the KT date as the index. While there is an ongoing debate about whether to use individual comorbidities individually in an analysis or a summary index such as the CCI [7], we opted for the latter, given the relatively small sample size. We categorized the CCI as no comorbidity (0), mild (1–2), moderate (3–4), and severe (≥ 5) following the literature (Additional file 1: Table S1) [8]. Other patient clinical characteristics were donor type (deceased versus a living donor), patient’s county, and graft status. Age, blood type, and donor type came from MOTP. Data on sex at birth and CCI came from the HDNS data repository (HDNS data analyst computed the CCI for each patient).

### Measuring health care costs

The primary outcome was the mean annual health care costs per patient. Before KT, health care costs included dialysis, inpatient, and insured physician services. Costs incurred during KT included the pre-and post-operative hospital stay, organ procurement cost, and medications. Post-KT costs included inpatient, insured physician services, and immunosuppressants (post-transplant medication costs). The amount paid to physicians by the Medical Services Insurance (MSI) plan for insured physician services for eligible NS residents from the MED database, Canadian Institute for Health Information (CIHI) (at the HDNS), served as a proxy for insured physician services. We calculated inpatient costs by multiplying the resource intensity weights (RIW), per patient, by the cost of a standard hospital stay for NS for 2019/20 of $6477 [9]. The RIW data came from the HDNS data repository.

We had data on dialysis type, start date, and KT date for patients on dialysis at the time of KT. Using the dialysis start date and transplantation date from the MOTP, we calculated the number of years on dialysis. However, there were no patient-level data for actual cost per visit to a dialysis centre. Hence, the annual dialysis cost data came from a Canadian study, Beaudry et al*.* [10]*.* Beaudry et al*.* conducted a descriptive cost analysis of the different dialysis treatment modalities from a Canadian payer health care system’s perspective in Manitoba, Canada. These costs included facility-based hemodialysis (HD), home HD, peritoneal dialysis (PD) and the training costs associated with PD and home HD. In addition, dialysis costs included direct expenses related to human resources—registered nurses, unit clerks, licensed practical nurses, dieticians, dialysis technicians, clinical pharmacists, and social workers. These direct costs also included benefits, vacation and relief, and costs associated with supplies, including medical, surgical, laboratory, housekeeping, and maintenance [10]. The costs also included drug, equipment, departmental sundry, overhead, water, capital, and in-centre run. The original amounts in Beaudry et al. were in 2016 dollars. However, we converted into 2019 dollars using the Canadian consumer price index (CPI) for health and personal care.

We included a one-time organ procurement cost of $26,943 per patient for the KT year, representing the in-country organ procurement cost specified in the April 2019 interprovincial billing rates for designated high-cost transplants of the Interprovincial Health Insurance Agreements Coordinating Committee (IHIACC) in Canada [11]. The understanding was that the amount represents the value of health resources used in procuring, storing, shipping and maintaining an organ, which includes the work of the coordinators and the specialists involved [11]. According to IHIACC, the organ procurement cost also includes the health care costs associated with maintaining the donor [11]. Consequently, we assumed the same organ procurement costs for deceased and living kidney donors. During a KT, patients spend days in the hospital for recovery, during which they are under observation. Therefore, costs during KT included costs associated with the period of monitoring and observation and the cost of dialysis for those experiencing delayed graft function.

Transplantations take place at the QEII Health Sciences Centre in Halifax, NS. All KT patients receive prophylaxis to reduce infectious complications at transplantation. These include anti-pneumocystis jirovecii pneumonia (PJP) prophylaxis (Septra or Pentamidine), anti-herpes simplex viruses (HSV) (Acyclovir or Valcyte), and anti-urinary tract infection (UTI) (Septra or Keflex). Patients at high risk also receive anti-cytomegalovirus (CMV) (Valcyte). The first agent listed in each case was the agent of choice unless contraindicated. In addition to infectious prophylactic medications, all KT patients receive induction therapy at transplantation. Patients with a high panel-reactive antibody (PRA) (highly sensitized) receive anti-thymocyte globulin (ATG); patients at lower risk get Basiliximab (BSX). KT patients also require maintenance immunosuppressive agents available in various combination regimens over time. We did not have patient-level medication cost data, so we sourced the medication costs per patient from a published Canadian study [12]. The medication costs varied by donor type. Medication costs associated with receiving an organ from a living kidney donor were $15,224 for the first year and $1953 for each subsequent year. For a deceased donor, costs were $14,405 for the first year and $2174 for each subsequent year.

Canada has a publicly funded health care system. In 2019, the public sector’s share of total health expenditure was 70% compared to 30% for the private sector [13]. The 70% public sector share for 2019 was below the average for countries in the Organisation for Economic Co-operation and Development (OECD), 73% [13]. The public–private split for 2019 for the United States (US) was 49% public, 51% private; the United Kingdom, 79% public, 21% private; and Germany, 85% public, 15% private [13]. Canada's health care is governed by the Canada Health Act, which defines the Canadian health insurance system [14]. The Canadian government, under the Act, sets out conditions for qualification for the federal Canada Health Transfer for the provinces and territories. The Act requires universal coverage for medically necessary health care and reasonable access to essential hospital and physician services for all insured persons based on need and not the ability to pay [14]. Therefore, the health care costs reported in this study closely approximate the actual care costs rather than prices or charges.

The provincial health insurance scheme covers all kidney transplantations for all NS patients with a valid NS health card. In addition, hospital visits and in-hospital medications are publicly insured, per the Canada Health Act. In addition, the province operates two drug insurance programs for out-of-hospital medications. First, there is Seniors' Pharmacare for Nova Scotians 65 years and over who do not have private coverage or coverage under any other program. Second, the Family Pharmacare Program is available to all Nova Scotians without drug coverage or those facing high drug costs. The high-cost drug program under the Family Pharmacare Program covers the cost of immunosuppressant medications.

All costs were in 2019 Canadian dollars. The purchasing power parity conversion between the Canadian dollar (CAD) and the US dollar (USD) for 2019 was 1 USD equals 1.213 CAD [15]. Since our goal in the study was to evaluate health care costs over time, patients with failed grafts remained in the analysis. However, we removed patients who died or moved out of the province from the analysis in subsequent periods [16, 17].

### Statistical analysis

We presented binary and categorical variables as percentages for the entire sample and stratified by donor type. We compared costs before to after KT. We used the mean of three years of cost data before KT and three years after KT to reflect post-KT costs. Also, we added KT-related costs to the post-KT period. We reported the mean and standard deviation (SD) and the median and interquartile range (IQR) or the 25th and 75th percentiles for the relevant variables. We reported cost differences and quantified the uncertainty around the estimates using 95% confidence intervals (CIs) constructed from non-parametric bias-corrected bootstrapping from 1000 replications based on sampling with replacement from the original data [18]. We performed additional sensitivity analysis by estimating cost savings using adjusted generalized linear models (GLM) with a log link function and a gamma distribution, clustering at the level of the patients’ county of residence. We constructed the 95% CIs from bootstrapped standard errors [19]. We adjusted for patients’ characteristics listed above. In a further sensitivity analysis, we compared costs across all three periods: before KT, KT year, and after KT, again, using an adjusted GLM, without adding KT-related costs to the post-KT period.

We used a semi-parametric finite mixture model (FMM) to identify data-driven cost groups using pre-transplantation annual inpatient costs. Conceptually, finite mixture models are probabilistic models that combine density functions and are based on a framework that treats observed data as coming from distinct but unobserved subpopulations [20, 21]. An FMM examines sub-groups within a given patient population without imposing pre-defined groups on the observed data [20, 21]. We did not know the number of latent cost groups a priori. Therefore, it was determined empirically with the Akaike information criterion (AIC) and Bayesian information criterion (BIC) model comparison criteria. In FMM, cost group membership depends on the posterior probability that a patient belongs to a particular group [22].

We estimated finite mixture models of GLM regressions with gamma densities and log links for pre-KT inpatient costs. We first estimated a two-component model (two groups) and the associated AIC and BIC. Then, we did the same for a three-component model (three groups) using Stata’s *fmm* module in both cases [21, 22]. Next, we compared the AIC and the BIC between the two specifications to determine the model that provided the best fit [21]. We then estimated the posterior probabilities of cost group membership after estimating the GLM model of the selected specification [21, 22]. These posterior probabilities provided a mechanism for assigning patients to cost groups [21]. Finally, after defining the cost groups, we compared the mean annual costs of patients in these cost groups and their characteristics using the Pearson chi-square.

## Results

### Study population

The total sample consisted of 340 ESKD patients in NS who received a single KT between January 1, 2011, and December 31, 2018. Out of these, 331 had complete cost data before and after KT; hence they were included in the primary analysis. Of the 331, a majority were males, had a CCI of ≤ 2, and had HD before KT. In addition, 81.6% received an organ from a deceased donor. The median age at transplantation was 53 years (IQR: 20), and the median years on dialysis was 1.9 years (IQR: 2.2) (Table 1). Recipients of a deceased donor kidney spend a median of 2.2 (IQR:2.4) years on dialysis compared to 1.3 (IQR: 1.1) for living donor kidney recipients (Table 1).

**Table 1:** Clinical and demographic characteristics of the study population

Variable	Total (N = 331)	Recipients of deceased donor kidney (n = 270; 81.6%)	Recipients of living donor kidney (n = 61; 18.4%)
Sex at birth			
Female	31.1%	30.7%	32.8
Male	68.9%	69.3%	67.2
Age at KT			
Mean (SD)	51.9 (14.0)	52.8 (13.6)	48.4 (14.6)
Median (IQR)	53.0 (20.0)	54 (20.0)	50 (22.0)
Charlson comorbidity index			
No comorbidity (0)	4.8%	4.8%	4.9%
Mild (1–2)	68.8%	68.2%	70.5%
Moderate (3–4)	20.6%	20.0%	23.0%
Severe (≥ 5)	5.8%	7.0%	1.6%
Dialysis type			
Hemodialysis	57.0%	57.4%	55.7%
Peritoneal dialysis	32.7%	31.4%	39.3%
Pre-emptive	10.3%	11.5%	4.9%
Blood type			
A	38.8%	36.3%	50.8%
AB	3.6%	4.1%	1.6%
B	12.1%	13.0%	8.2%
O	45.5%	46.7%	39.3%
Years on dialysis			
Mean (SD)	2.5 (2.0)	2.7 (2.1)	1.6 (1.2)
Median (IQR)	1.9 (2.2)	2.2 (2.4)	1.3 (1.1)

*SD* standard deviation, *IQR* interquartile range, *KT* kidney transplantation

The three-component FMM model had the lowest AIC and BIC compared to the two-component model, suggesting a better fit. We labelled the groups based on their mean health care costs; high-cost (HC), medium-cost (MC) and low-cost (LC) groups. Approximately 3% of patients were in the LC group, 52% in the MC group and 45% were in the HC group. Cost group membership was the same before and after KT. Patients in the cost groups differed by CCI category and graft status. Patients in the HC group had a greater comorbidity burden characterized by CCI ≥ 3 and a higher proportion of graft failure. They also spent more time on the waitlist than those in the LC and MC groups (Table 2).

**Table 2:** Patient characteristics by cost group

	Low cost (2.9%)	Medium cost (51.8%)	High cost (45.3%)	P-value
Sex at birth				0.638
Female	18.2%	31.4%	32.0%	
Male	81.2%	68.6%	68.0%	
Charlson comorbidity index				< 0.001
No comorbidity (0)	–	4.9%	5.2%	
Mild (1–2)	72.7%	74.0%	55.7%	
Moderate (3–4)	27.3%	18.4%	24.7%	
Severe (≥ 5)	–	2.7%	14.4%	
Dialysis type				0.459
Hemodialysis	45.5%	54.7%	57.1%	
Peritoneal dialysis	36.4%	34.1%	32.6%	
Not on dialysis	18.2%	11.2%	10.3%	
Blood type				0.711
A	36.4%	38.1%	41.2%	
AB	–	4.9%	1.0%	
B	9.1%	12.1%	12.4%	
O	54.6%	44.8%	45.4%	
Organ type				0.696
Deceased donor	90.1%	81.6%	80.4%	
Live donor	9.1%	18.4%	19.6%	
Transplant status				0.042
Functioning graft	100%	94.2%	86.6%	
Graft failed	-	5.8%	13.4%	
Age at transplantation				-
Median (IQR)-years	60 (22)	51 (21)	57 (19)	
Mean (SD)	56.1 (13.4)	50.1 (13.9)	55.8 (13.1)	
Number of years on dialysis				-
Median (IQR)-years	1.7 (1.5)	1.7 (2.3)	2.2 (2.3)	
Mean (SD)	2.0 (0.4)	2.4 (0.2)	2.7 (0.3)	

We used a semi-parametric three-component finite mixture model to identify these data-driven cost groups based on patients' pre-transplantation annual inpatient costs. *SD* standard deviation, *IQR* interquartile range

### Annual health care costs and savings

The mean annual cost per patient before KT was $78,661 (SD: $32,029), which decreased to $59,071 (SD: $44,809) after KT (Table 3). Recipients of kidneys from deceased donors had a mean annual health care cost of $78,295 (SD: $32,912) before KT and $59,564 (SD: $47,161) after KT. The corresponding amounts for kidney recipients from living donors were $80,279 (SD: $27,975) and $56,891 (SD: $32,640). There were also decreases in annual insured physician services after KT (Table 3). Annual health care costs before KT varied by dialysis type. The annual health care costs before KT for patients on HD was $95,627 (95% CI: $92,375, $98,880), $64,985 (95% CI: $62,204, $67,766) for patients on PD, and $30,763 (95% CI: $25,674, $35,852) for patients not on dialysis. Table S3 shows the mean annual health care costs for each of the three years before and after KT. For example, two years before KT, the mean annual health care costs ranged from $72,684 to $81,636. In the KT year, the cost ranged from $111,185 to $122,990. However, two years after KT, annual health care costs decreased to between $21,232 and $26,666 (Additional file 1: Table S3).

**Table 3:** Mean and median annual health care costs per patient before and after kidney transplantation

Period and cost item	Full sample		Recipients of deceased donors' kidney		Recipients of living donors' kidney	
	Mean (SD)	Median (p25, p75)	Mean (SD)	Median (p25, p75)	Mean (SD)	Median (p25, p75)
Annual total health care costs						
Before transplantation	$78,661 ($32,029)	$79,466 ($59,164, $93,094)	$78,295 ($32,912)	$79,112 ($59,422, $92,769)	$80,279 ($27,975)	$81,863 ($59,155, $94,617)
After transplantation	$59,071 ($44,809)	$47,894 ($38,029, $63,353)	$59,564 ($47,161)	$47,861 ($38,182, $62,785)	$56,891 ($32,640)	$48,209 ($37,324, $66,248)
Annual insured physician services						
Before transplantation	$4639 ($4639)	$2742 ($935, $6330)	$4905 ($5873)	$3019 ($1069, $6677)	$3460 ($4527)	$1907 ($579, $4432)
After transplantation	$3786 ($3824)	$2757 ($1739, $4079)	$3880 ($3701)	$2838 ($1850, $4114)	$3373 ($4338)	$2045 ($1413, $3425)
Annual hospitalization costs						
Before transplantation	$23,563 ($21,978)	$18,092 ($11,592, $27,420)	$23,297 ($22,655)	$16,974 ($11,448, $27,099)	$24,744 ($18,812)	$20,765 ($13,760, $28,394)
After transplantation	$25,986 ($37,001)	$17,694 ($10,116, $28,183)	$26,291 ($39,960)	$16,955 ($9815, $27,870)	$24,632 ($19,200)	$21,642 ($15,519, $28,394)

*SD* standard deviation, *p25* 25th percentile, *p75* 75th percentile

The estimated mean annual cost savings per patient was $19,589 (95% CI: $14,013, $23,397), representing a 25% decrease in costs (Table 4). The percent decrease from the sensitivity analysis, which involved an adjusted GLM model, was $21,484 (95% CI: $14,935, $28,032), corresponding to a 27% decrease (Additional file 1:Table S4). The percent decrease in costs for recipients of kidneys from deceased donors was 24% versus a 29% decrease for recipients of kidneys from living donors. There was no statistically significant difference in inpatient costs before and after KT. However, recipients of deceased donors’ kidneys had a $390 to $1640 decrease in annual insured physician services after KT (Table 4). When we compared costs across all three periods: mean annual costs before KT, KT year, and after KT, we found that the treatment of KT-related costs in the analysis affected the magnitude of the cost savings. For example, when we compared post-KT to pre-KT costs without accounting for KT-related costs, we found a 60% decrease in mean annual health care costs (Additional file 1: Table S5).

**Table 4:** Mean annual health care cost savings per patient stratified by organ source

Cost	Full sample	Recipients of deceased donors' kidney	Recipients of living donors' kidney
	Mean annual cost savings after transplantation (95% CI) ^¥^	Mean annual cost savings after transplantation (95% CI) ^¥^	Mean annual cost savings after transplantation (95% CI) ^¥^
Annual total health care costs	$19,589 ($14,013, $23,397)	$18,731 ($12,118, $22,811)	$23,388 ($14,557, $32,556)
Annual insured physician services	$856 ($263, $1526)	$1025 ($390, $1640)	$88 (−$1616, $1550)
Annual hospitalization costs	− $2422 (− $6655, $511)	− $2995 (− $8141, $522)	$112 (− $5507, $6299)

^¥^ 95% confidence interval (CI) constructed from bootstrapping with replacement

### Cost groups

We identified the three patient cost groups using pre-KT annual inpatient costs and a three-component FMM. Patients with higher (lower) annual health care costs before KT also had higher (lower) costs after KT. Patients in the LC group had a mean annual cost of $51,240 (SD: $25,792), $71,840 (SD: $22,835) for MC, and $97,450 (SD: $41,236) for the HC group, all before KT (Table 5). The trend continued in the post-KT period (Table 5). In the case of patients in the LC group, there was no statistically significant difference in costs before and after KT, although their costs were lower after KT (Table 5). The HC group had the largest cost savings when comparing costs within each cost group before and after KT (Table 5). KT was associated with a 24% decrease for the MC group and 26% for the HC group. When we compared costs in each group to the corresponding mean for the entire sample, patients in the HC group had 24% higher mean annual health care costs before KT than the corresponding mean for the total sample, while the LC group had 35% lower costs.

**Table 5:** Mean annual health care costs and cost savings by cost groups

Period	Low-cost group	Medium-cost group	High-cost group
	Mean (SD)	Mean (SD)	Mean (SD)
Full sample			
Before KT	$51,240 ($25,792)	$71,840 ($22,835)	$97,450 ($41,236)
After KT	$42,400 ($14,828)	$54,318 ($39,739)	$71,890 ($54,560)
Mean annual cost savings per patient, (95% CI)	$8839 (−$7719, $19,582)	$17,523 ($10,319, $22,072)	$25,560 ($16,570, $33,214)
Recipients of a deceased donor kidney			
Before KT	$55,618 ($22,468)	$71,316 ($24,029)	$97,488 ($42,579)
After KT	$41,178 ($15,034)	$53,995 ($42,125)	$74,915 ($56,716)
Mean annual cost savings per patient, (95% CI)	$14,440 ($4374, $21,005)	$17,321 ($8986, $22,813)	$22,573 ($12,940, $30,709)
Recipients of a living donor kidney			
Before KT	–	$74,171 ($16,573)	$97,294 ($36,238)
After KT	–	$55,749 ($27,060)	$59,473 ($43,746)
Mean annual cost savings per patient, (95% CI)	–	$18,421 ($10,664, $25,597)	$37,820 ($15,092, $57,949)

*KT* kidney transplantation, *SD* standard deviation, 95% confidence intervals (CI) constructed from bootstrapping with replacement

## Discussion

This study sought to quantify the mean annual cost savings associated with KT and identify and characterize patients in data-driven cost groups from a Canadian payer perspective. We quantified the cost savings per cost group using a novel approach to divide patients into cost groups based on their pre-KT annual inpatient costs. We found that KT was associated with annual cost reductions per patient, at least in the short term. Further, we did not find differences in inpatient costs before and after KT. However, costs associated with insured physician services differed. We empirically identified three cost groups in the patient population: high, medium, and low, with differences in mean annual health care costs and patient characteristics. After KT, the most significant cost savings were for patients in the high-cost group before KT. The results based on the defined cost groups were internally consistent. For example, although we used pre-KT inpatient costs in determining the cost groups, patients in the low-cost group before KT also had lower annual health care costs after KT. We observed and reported similar results for those in medium and high-cost groups. However, our analysis could benefit from more validation studies.

There is heterogeneity in studies directly estimating cost savings associated with KT, complicating comparing our results to previous studies. Existing studies differ in time horizons and their treatment of costs incurred during the year of KT. For example, Helanterä et al. [1] reported a 33% cost decrease comparing the first post-KT year to pre-KT. If we were to follow their approach, the equivalent reported cost savings in the present study would be 53% (Additional file 1: Table S3).

On the other hand, Jarl et al*.* [2] reported a 66–79% decrease in mean annual costs following KT. However, they followed patients ten years before and after KT. They assumed that health care costs in the absence of KT would remain the same for each patient on dialysis for ten years, which also implicitly assumes that the comparator, a dialysis patient, would be alive after ten years. While we used a different approach than the studies mentioned above, our findings were consistent with other cost studies conducted in Canada. For example, according to Klarenbach et al*.* [23], the annual health care costs of treating ESKD patients on in-centre HD ranged from $101,004 to $113,763; home HD: $75,487 to $95,688; and PD $59,539, all measured in 2019 dollars. The current study found comparable annual health care costs before transplantation; in-centre HD: $95,265 to $102,277, home HD: $67,496 to $78,134, PD: $62,204 to $67,766 and those not on dialysis: $25,674 to $35,852, all in 2019 dollars.

Whether KT contributes to decreases in health care costs depends on the comparator, time horizon, and the treatment of costs incurred in the KT year. KT recipients live longer compared to patients on dialysis. However, that longevity complicates the link between receiving a KT and health care costs. Our study focussed on short-term cost savings. Therefore, we used at most three years on either side of KT. We demonstrated that costs were lower in the post-KT period despite this approach. As we showed, the cost savings were much higher when we excluded the costs incurred in the KT year and compared the three-year averages before and after KT. The cost savings also depend on the cost of immunosuppressants. Decreases in the costs of immunosuppression agents will increase the costs savings associated with KT, irrespective of the time horizon.

The current study results will allow stakeholders in NS in the context of the deemed consent legislation and elsewhere to set reasonable targets for judging KT's economic impact and identifying factors that may impact the magnitude of cost savings associated with KT. In addition, information about the potential cost savings per patient can be combined with estimates of changes in transplantation activity to better anticipate the cost savings for the health system.

### Limitations

The magnitude of the potential cost savings will depend on the study's perspective. Perspective speaks to whose costs were included in the analysis. The current study was from a Canadian single-payer perspective for two reasons. The first reason was data limitations. Unlike a societal perspective, a payer perspective includes only costs incurred by the formal health care sector. A societal perspective consists of all costs irrespective of who incurs them. These include patients' out-of-pocket expenses (for visits to the dialysis centre) and informal health sector costs. The informal health sector costs include patient-time, unpaid caregiver-time, transportation, social services, and non-health sector costs such as lost labour market earnings and uncompensated household production [24]. Since we used existing data, we did not have all the data required for a societal perspective. Second, the literature suggests that the adopted perspective depends on the context and the research question [24]. We were reasonably confident that a payer perspective was adequate given our study's objective. Therefore, the results reported in this study are likely to be conservative estimates because the actual cost savings will most likely be higher if measured from a societal perspective.

Also, the current study was based on non-experimental real-world data. However, the nature of the patient population makes it impractical to conduct a meaningful and ethical randomized control trial. Accordingly, we took steps to reduce threats to the internal validity of the results. Besides each patient serving as their control, we limited the sample to three years of data on either side of KT to reduce potential threats to internal validity. These threats include history, instrumentation, and maturation [25]. In history, events external to the intervention could affect health care costs. The more extended the period between the pre-post periods, the more likely history will be an issue. Limiting the before-after period to 3 years most likely minimized this threat. In instrumentation, changes in valuing health resources over time could affect outcomes. But this threat was unlikely to be a significant issue since we measured all costs in constant 2019 dollars, thereby eliminating the potential effects of inflation on measured costs. Finally, in the case of maturation, variables that change with time, like ageing, could potentially affect health care costs. Comparing costs of patients as their own controls over a limited time window minimizes the maturation threat to internal validity.

We sourced medication cost data from a Canadian study. These costs were initially higher in the first year, decreased over time, and varied by donor type. The medication costs may vary somewhat at the patient level, but given the standardization of the treatment protocols in Canada, any difference in medication costs is likely to be small and would not change the conclusions. In addition, we quantified the uncertainty around the cost savings by reporting 95% CIs from non-parametric and parametric bootstrapping where appropriate.

Also, we used the nationally reported organ procurement rate as a proxy for kidney procurement costs. The organ procurement rate reported in the Interprovincial Health Insurance Agreements Coordinating Committee in Canada included all costs associated with acquiring, storing, maintaining, and transporting an organ. It also included the hospital costs of maintaining the donor, including living donors. Most organ retrievals and all transplantations occur at the QEII in Halifax, so, from that perspective, the transportation cost component may likely be lower. At the same time, the MOTP also receives organs transported into Halifax from other provinces. Given the standardization of organ retrieval protocols across the country, the magnitude of organ procurement cost differences is unlikely to change our conclusions. However, future studies should consider how organ procurement costs could impact the results.

## Conclusions

The current study demonstrated that KT was associated with a 24–29% decrease in mean annual health care costs per patient in the short term. We also showed that high-cost patients tend to spend more time on the waitlist. Therefore, successful implementation of the deemed consent legislation could reduce the number of years on the waitlist, reducing health care costs per patient, at least in the short term.

## Supplementary Information

**Additional file 1: Table S1.** Charlson Comorbidity Index (CCI) ICD-10 comorbidities. **Table S2.** ICD-10 and CCI codes used to identify patient and cost elements. **Table S3.** Summary statistics of total health care costs over time stratified by organ source. **Table S4.** Sensitivity analysis: Adjusted mean annual health care cost savings per patient stratified by donor type. **Table S5.** Differences in mean annual health care costs per patient before, during, and after KT from a GLM model.

## Data Availability

The raw data on health resource utilization that supports the findings of this study are available from Health Data Nova Scotia. However, restrictions apply to the availability of the data. After a data access request approval, we obtained access to the data, which is not publicly available. We displayed all other data in the manuscript.
