# Supplementary material for: What are the short-term annual cost savings associated with kidney transplantation?
Source: Cost Eff Resour Alloc. 2022 May 3;20:20. doi: 10.1186/s12962-022-00355-2 (PMC9063122; doi:10.1186/s12962-022-00355-2)
Supplement: Supplementary file 1 — Additional file 1: Table S1. Charlson Comorbidity Index (CCI) ICD-10 comorbidities. Table S2. ICD-10 and CCI codes used to identify patient and cost elements. Table S3. Summary statistics of total health care costs over time stratified by organ source. Table S4. Sensitivity analysis: Adjusted mean annual health care cost savings per patient stratified by donor type. Table S5. Differences in mean annual health care costs per patient before, during, and after KT from a GLM model. [file 12962_2022_355_MOESM1_ESM.docx]

Table S1. Charlson Comorbidity Index (CCI) ICD-10 comorbidities

| Medical condition | ICD-10 | Comorbidity weight |
| --- | --- | --- |
| Myocardial Infarction | I21.^, I22.^, 125.2 | 1 |
| Congestive Heart Failure | I09.9, I11.0, I13.0, I13.2, I25.5, I42.0, I42.5-I42.9, I43.^, I50.^, P29.0 | 1 |
| Peripheral Vascular Disease | I70.^, I71.^, I73.1, I73.8, I73.9, I77.1, I79.0, 179.2, K55.1, K55.8, K55.9, Z95.8, Z95.9 | 1 |
| Cerebrovascular Disease | G45.^, G46.^, H34.0, I60.^-I69.^ | 1 |
| Dementia | F00.^-F03.^, F05.1, G30.^, G31.1 | 1 |
| Chronic Pulmonary Disease | I27.8, I27.9, J40.^-J47.^, J60.^-J67.^, J68.4, J70.1, J70.3 | 1 |
| Rheumatic Disease | M05.^, M06.^, M31.5, M32.^-M34.^, M35.1, M35.3, M36.0 | 1 |
| Peptic Ulcer Disease | K25.^-K28.^ | 1 |
| Mild Liver Disease | B18.^, K70.0-K70.3, K70.9, K71.3-K71.5, K71.7, K73.^, K74.^, K76.0, K76.2-K76.4, K76.8, K76.9, Z94.4 | 1 |
| Diabetes without Chronic Complications | E10.0, E10.1, E10.6, E10.8, E10.9, E11.0, E11.1, E11.6, E11.8, E11.9, E12.0, E12.1, E12.6, E12.8, E12.9, E13.0, E13.1, E13.6, E13.8, E13.9, E14.0, E14.1, E14.6, E14.8, E14.9 | 1 |
| Diabetes with Chronic Complications | E10.2-E10.5, E10.7, E11.2-E11.5, E11.7, E12.2-E12.5, E12.7, E13.2-E13.5, E13.7, E14.2-E14.5, E14.7 | 2 |
| Hemiplegia or Paraplegia | G04.1, G11.4, G80.1, G80.2, G81.^, G82.^, G83.0-G83.4, G83.9 | 2 |
| Renal Disease | I12.0, I13.1, N03.2-N03.7, N05.2-N05.7, N18.^, N19.^, N25.0, Z49.0-Z49.2, Z94.0, Z99.2 | 2 |
| Any malignancy including Lymphoma and Leukemia except Malignant Neoplasm of Skin | C00.^-C26.^, C30.^-C34.^, C37.^-C41.^, C43.^, C45.^-C58.^, C60.^-C76.^, C81.^-C85.^, C88.^, C90.^-C97.^ | 2 |
| Moderate or Severe Liver Disease | I85.0, I85.9, I86.4, I98.2, K70.4, K71.1, K72.1, K72.9, K76.5, K76.6, K76.7 | 3 |
| Metastatic Solid Tumor | C77.^-C80.^ | 6 |
| AIDS/HIV | B20.^-B22.^, B24.^ | 6 |

# ICD -10, International Statistical Classification of Diseases and Related Health Problems 10^th^

# Revision; CCI, Canadian Classification of Health Interventions.

Table S2. ICD-10 and CCI codes used to identify patient and cost elements

| Condition/Procedure | ICD-10 and CCI codes |
| --- | --- |
| End-stage kidney disease | N18.6 |
| Dependence on renal dialysis | Z99.2 |
| Kidney transplant status | Z94. 0 |
| Encounter for aftercare following a kidney transplant | Z48.22 |
| Kidney transplant rejection | T86.11 |
| Kidney transplant failure | T86.12 |
| Kidney transplant infection | T86.13 |
| Other complication of a kidney transplant | T86.19 |
| Kidney dialysis | 1.PZ.21. ^^ |
| Hemodialysis | 1.KY.76. ^^ |
| Peritoneal (for dialysis) / Peritoneal cavity | 1.OT.53. ^^ |
| Shunt (renal dialysis)/Renal dialysis) | 1.KY.54. ^^ |
| Kidney transplantation | 1.PC.85. ^^ |

# ICD -10, International Statistical Classification of Diseases and Related Health Problems 10^th^

# Revision; CCI, Canadian Classification of Health Interventions.

Table S3. Summary statistics of total health care costs over time stratified by organ source

| Period | Full sample  Mean  (95% CI) | Recipients of deceased  donors' kidney  Mean  (95% CI) | Recipients of  living  donors' kidney  Mean  (95% CI) |
| --- | --- | --- | --- |
| Three years before KT transplantation | $76647  ($71550, $81745) | $77269  ($71459, $83079) | $73208  ($64388, $82021) |
| Two years before KT transplantation | $77160  ($72684, $81636) | $77517  ($72525, $82509) | $74517  ($67433, $81601) |
| One year before KT transplantation | $82052  ($77702, $86403) | $81636  ($76722, $86551) | $84185  ($75374, $92997) |
| KT transplantation year | $117087  ($111185, $122990) | $118053  ($111300, $124806) | $11918  ($101897, $121940) |
| One year after KT transplantation | $38223  ($34334, $42113) | $38781  ($34128, $43434) | $35590  ($32029, $39154) |
| Two years after KT transplantation | $23949  ($21232, $26666) | $24743  ($21636, $27850) | $19205  ($15645, $22764) |
| Three years after KT transplantation | $23749  ($20926, $26572) | $24677  ($21371, $27983) | $19395  ($15353, $23438) |

KT, kidney transplantation; CI, confidence interval

Table S4. Sensitivity analysis: Adjusted mean annual health care cost savings per patient stratified by donor type

| Cost | Full sample | Recipients of deceased  donors' kidney | Recipients of living  donors' kidney |
| --- | --- | --- | --- |
|  | Mean annual cost savings after transplantation  (95% CI) | Mean annual cost savings after transplantation  (95% CI) | Mean annual cost savings after transplantation  (95% CI) |
| Annual total health care costs | $21484  ($14935, $28032) | $20885  ($13809, $27960) | $25534  ($18982, $32086) |
| Annual insured physician services | $1190  ($583, $1796) | $1257  ($682, $1833) | $614  (-$607, $1835) |
| Annual hospitalization costs | -$827  (-$4312, $2657) | -$1405  (-$5297, $2487) | $2796  (-$1480, $7071) |

The results reported here came from an adjusted generalized linear model (GLM). We adjusted for sex at birth, blood type, organ type (organ from a deceased versus a living donor), dialysis type, age at transplantation and age squared. The 95% CI came from cluster robust standard errors, clustering at the level of the patient county with bootstrapping.

Table S5. Differences in mean annual health care costs per patient before, during, and after KT from a GLM model

| Sample | Before KT  Mean cost per year  (95% CI) | KT year  Mean  (95% CI) | After KT  Mean cost per year  (95% CI) | Difference  (95% CI)  (KT year – Before KT) | Difference  (95% CI)  (Before – After KT) |
| --- | --- | --- | --- | --- | --- |
| Full sample | $78999  ($76331, $81668) | $117087  ($111185, $122990) | $29313  ($27347, $31278) | $39633  ($35864, $43402) | $47374  ($39008, $55741) |
| Donor type | | | | | |
| Recipients of deceased  donors' kidney | $79079  ($76077, $82081) | $118053  ($111301, $124805) | $29993  ($27695, $32291) | $40235  ($35372, $45098) | $46931  ($38096, $55767) |
| Recipients of living  donors’ kidney | $78533  ($73455, $83611) | $111918  ($101931, $121906) | $25864  ($23353, $28375) | $34819  ($25199, $44439) | $50704  ($42932, $58477) |

We adjusted for sex at birth, blood type, organ type (organ from a deceased versus a living donor), dialysis type, age at transplantation and age squared. The 95% CI came from cluster robust standard errors, clustering at the level of the patient county with bootstrapping. KT, kidney transplantation; GLM, generalized linear model.
